# Gut microbiota determines the social behavior of mice and induces metabolic and inflammatory changes in their adipose tissue

**DOI:** 10.1038/s41522-021-00193-9

**Published:** 2021-03-19

**Authors:** Oryan Agranyoni, Sapir Meninger-Mordechay, Atara Uzan, Oren Ziv, Mali Salmon-Divon, Dmitry Rodin, Olga Raz, Igor Koman, Omry Koren, Albert Pinhasov, Shiri Navon-Venezia

**Affiliations:** 1 Department of Molecular Biology, Faculty of Natural Sciences, Ariel University, Ariel, Israel; 2 Azrieli Faculty of Medicine, Bar-Ilan University, Safed, Israel; 3 The Dr. Miriam and Sheldon G. Adelson School of Medicine, Ariel University, Ariel, Israel

**Keywords:** Microbiome, Next-generation sequencing

## Abstract

The link between the gut microbiota and social behavior has been demonstrated, however the translational impact of a certain microbiota composition on stable behavioral patterns is yet to be elucidated. Here we employed an established social behavior mouse model of dominance (Dom) or submissiveness (Sub). A comprehensive 16S rRNA gene sequence analysis of Dom and Sub mice revealed a significantly different gut microbiota composition that clearly distinguishes between the two behavioral modes. Sub mice gut microbiota is significantly less diverse than that of Dom mice, and their taxa composition uniquely comprised the genera *Mycoplasma* and *Anaeroplasma* of the Tenericutes phylum, in addition to the Rikenellaceae and Clostridiaceae families. Conversely, the gut microbiota of Dom mice includes the genus *Prevotella* of the Bacteriodetes phylum, significantly less abundant in Sub mice. In addition, Sub mice show lower body weight from the age of 2 weeks and throughout their life span, accompanied with lower epididymis white adipose tissue (eWAT) mass and smaller adipocytes together with substantially elevated expression of inflammation and metabolic-related eWAT adipokines. Finally, fecal microbiota transplantation into germ-free mice show that Sub-transplanted mice acquired Sub microbiota and adopted their behavioral and physiological features, including depressive-like and anti-social behaviors alongside reduced eWAT mass, smaller adipocytes, and a Sub-like eWAT adipokine profile. Our findings demonstrate the critical role of the gut microbiome in determining dominance vs. submissiveness and suggest an association between gut microbiota, the eWAT metabolic and inflammatory profile, and the social behavior mode.

## Introduction

Gut microbiota is increasingly recognized as a potential shaper of brain functions. This effect is mediated by the gut–brain axis^1^ through a bi-directional cross-talk^2^ that involves metabolic^3^, nutritional^4^, endocrine^5^, and immunological aspects^6,7^. Alterations in the microbiome–gut–brain axis are involved not only in the development of pathologies of the central nervous system^8,9^ and behavioral disorders^1,10^ but also in the regulation of social behavior^11,12^. An important aspect of social behavior is social interactions, which play a fundamental role in daily life^13^, highly influence well-being and quality of life, and often trigger the progression of various pathologies, including, but not limited to, metabolic^10,14^ and psychiatric disorders^11,12,15,16^.

A range of microbiome-related effects on social behavior has been reported^17–19^. Bacterial transplantation from specific pathogen-free to germ-free (GF) mice increased the sociability of the latter^20–22^, while social defeat, which leads to anxiety- and depressive-like behaviors, has been correlated with changes in the overall diversity of mouse gut microbiome and with a decrease in the relative abundance of specific bacterial genera^23,24^. In addition, chronic mild stress altered gut microbiota and affected mouse sociability^19^^[,21,23^, and recent studies suggest that prebiotics reduce anxiety-like behavior and improve social behavior in rodents, which was accompanied by changes in microbiota composition^25^.

Fecal transplantation studies in GF animals suggest that the gut microbiota may play a causal role in the development of chronic inflammation, which may be manifested in adipose tissue pathologies^26,27^. Recently, Oddy et al. described an association between adipose tissue-related inflammation and mental disturbances, including depression^28^. However, studies that directly link the gut microbiome, adipose inflammation and behavior are lacking. Thus, herein by using a well-defined mouse model of social dominance and submissiveness, we aimed to evaluate the above-mentioned inter-relations. These dominant and submissive mice were developed from a Sabra mouse lineage (HsdHu:SABRA-M, ENVIGO, Israel) using a selective breeding approach based on a food competition social interaction dominant–submissive relationship (DSR) test^29,30^. These mice showed strong and stable behavioral characteristics of either dominance or submissiveness, which represent important elements of the social behavioral spectrum^30,31^. Using comprehensive behavioral and pharmacological approaches^29,32^, we previously demonstrated that dominant (Dom) and submissive (Sub) mice differentially respond to psychotropic agents^29^ and stressogenic^30^, possess different life spans^33^, distinct brain neurochemistry^34^, and cognitive and learning capabilities^35^. More specifically, while Dom mice are relatively stress resilient, Sub mice exhibit strong sensitivity to stress^30^, alongside depressive-like and anti-social^30^ characteristics^36^, as well as systematic inflammation, demonstrated by higher interleukin (IL)-6 and IL-1b serum levels^33^. The multi-aspect differences between Dom and Sub animals led us to hypothesize that these mice possess different gut microbiome compositions, which may play a crucial role in shaping their social behavior by inducing inflammation leading to alterations in adipose tissue physiological homeostasis.

To test this hypothesis, we determined the gut microbiota composition of adult Dom and Sub mice and analyzed various aspects of their epididymis white adipose tissue (eWAT). Moreover, through fecal microbiota transplantations (FMTs) from Dom or Sub mice to GF mice, we demonstrate the critical role of the gut microbiome in shaping social behavioral modes. These gut microbiome-induced behavioral alterations were accompanied by FMT-driven changes in eWAT metabolism and inflammatory markers, which provides new insights regarding the determinants of social behavior.

## Results

### Dom and Sub mice exhibit different social behavior patterns and body weights

**Fig. 1:**
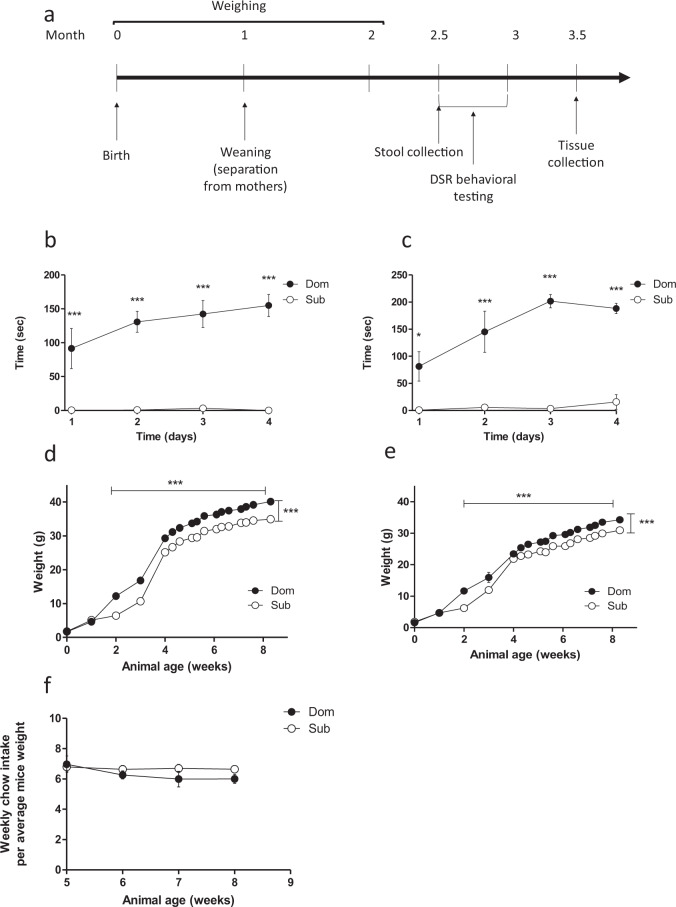
Sub mice demonstrate a more submissive social behavior and weigh less than Dom mice, despite similar food intake. **a** Experimental timeline. Dom and Sub mice were weighed from birth to 2 month old, then Dom and Sub mice behavior was assessed using the DSR test, followed by stool collection and tissue collection. **b**, **c** The dominant–submissive relationship (DSR) test results of Dom and Sub male (**b**) and female (**c**) mice (*n* = 10 in each group). The typical social behavior of each mouse was determined by measuring the average drinking time spent by the respective mouse group. **d**, **e** Body weight monitoring of Dom and Sub male (**d**) and female (**e**) mice. Mice of each behavioral phenotype were weighed three times a week and the average body weights are shown. **f** The chow intake by Dom and Sub mice (males and females combined) determined in parallel to weight follow-up. Each time point represents the average chow intake in eight cages per group, normalized to the average weight of mice housed in the respective cages. Weight and chow intake differences were statistically analyzed by using a two-way ANOVA followed by Bonferroni correction. ***p < 0.001. Error bars show standard deviation.

The social behavior of Sub mice was more submissive than that of Dom mice, independent of sex (Fig. 1b, c). These differences were observable from the second day of the DSR test. It is a social food competition behavioral test between a pair of mice, which are placed in the same apparatus after an overnight fasting. The drinking duration time of each mouse is measured for a period of 5 min during 4 consecutive days. In this test, the mouse that spends longer time drinking the milk compared to its counterpart is defined as “dominant” (Dom) and its pair mouse is defined as “submissive” (Sub). In this study, offspring of generation F24–F26 of selectively bred dominant and submissive mice were used. As demonstrated in Fig. 1, the clear Dom–Sub relationships (represented by the drinking time) are being established between the two behavioral groups (Dom and Sub males SD = 27.08 and 3.26 s, respectively, and Dom and Sub female SD = 13.71 and 0.72 s, respectively). The body weight of all mice constantly increased from the age of 2 weeks to the age of 8 weeks, but the average body weight of Dom mice was significantly (1.15-fold) higher than that of Sub mice throughout the growth period (Fig. 1d, e, *p* < 0.001). Although males weighed significantly more than females within each behavioral group (Weights at 8 weeks were: Dom: males, 39.9 ± 2.27 g, females, 34.2 ± 1.96 g; Sub: males, 34.9 ± 2.12 g, females, 30.9 ± 1.7 g; *p* < 0.001), the weight differences between Dom and Sub mice were sex independent (Fig. 1d, e). Food intake was similar in Dom and Sub mice (*p* > 0.05; Fig. 1f), indicating that the observed body weight differences were not due to differences in feeding behavior.

### Sub mice contain lower eWAT mass and smaller adipocytes

**Fig. 2:**
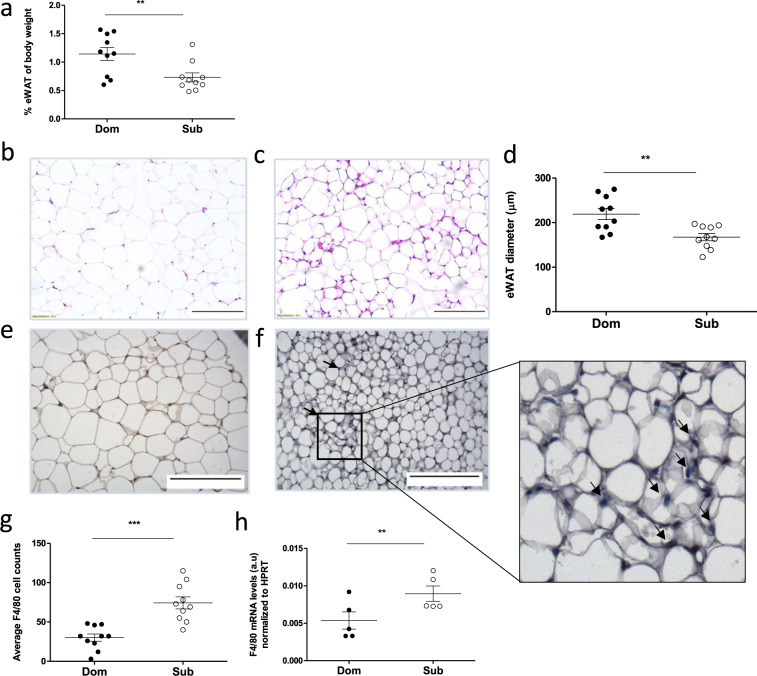
Sub mice show lower eWAT mass, smaller adipocytes, and increased macrophage infiltration, as compared with Dom mice. **a** Quantification of eWATs obtained from naive Dom and Sub mice (*n* = 10 in each group), normalized to the body weight of the respective mouse. Dom mice exhibit higher epididymis adipose mass. **b**, **c** Microscopic visualization of histological staining (×40 magnification, scale bar = 250 μm) of eWAT removed from Dom (**b**) and Sub (**c**) mice. **d** Quantification of adipocyte diameter of Dom and Sub mice determined from histological images using the ImageJ software. eWAT cells from 10 random cells per field were analyzed from 10 random fields in each mouse (SD of the diameter = 6.447 and 4.853 μm for Dom and Sub mice, respectively). The morphological differences of adipose tissue in the two groups of mice can be seen. **e**–**g** Macrophage infiltration was assessed by using immunohistochemistry (×40 magnification) for F4/80 in Dom (**e**) and Sub (**f**) mice (*n* = 10 each), and it was quantified **g** from histological images by using the ImageJ software. Ten random cells per field were analyzed from 10 random fields in each mouse. Arrows in **f** indicate crown-like structures of macrophages between adipocytes. **h** Macrophage quantification using F4/80 mRNA gene expression, normalized to HPRT, showing an increase in macrophage expression in Sub mice. a.u: arbitrary units. Statistical significance was assessed using Student’s *t* test. **p < 0.01, ***p < 0.001. Error bars show standard deviation.

The eWAT content was 1.5-fold lower in Sub mice than in Dom mice (Fig. 2a, *p* < 0.01). Histological analyses revealed pronounced differences in eWAT morphology between Dom (Fig. 2b) and Sub (Fig. 2c) mice, and a quantitative analysis revealed a significant, 1.25-fold decrease in adipocyte size (*p* < 0.05, Fig. 2d) and a high cell size heterogeneity in Sub mice. Hematoxylin and eosin (H&E) stains and immunohistochemistry for F4/80 in the eWAT of Sub mice revealed crown-like structures, which are typical to macrophage infiltration. Indeed, the eWAT of Sub mice showed a 2.1-fold increase in macrophage infiltration (Fig. 2e–g, *p* < 0.001) and a 1.6-fold increase in F4/80 gene expression (Fig. 2h, *p* < 0.05), as compared with Dom mice.

### The eWAT of Sub mice exhibits higher adipokine levels

**Fig. 3:**
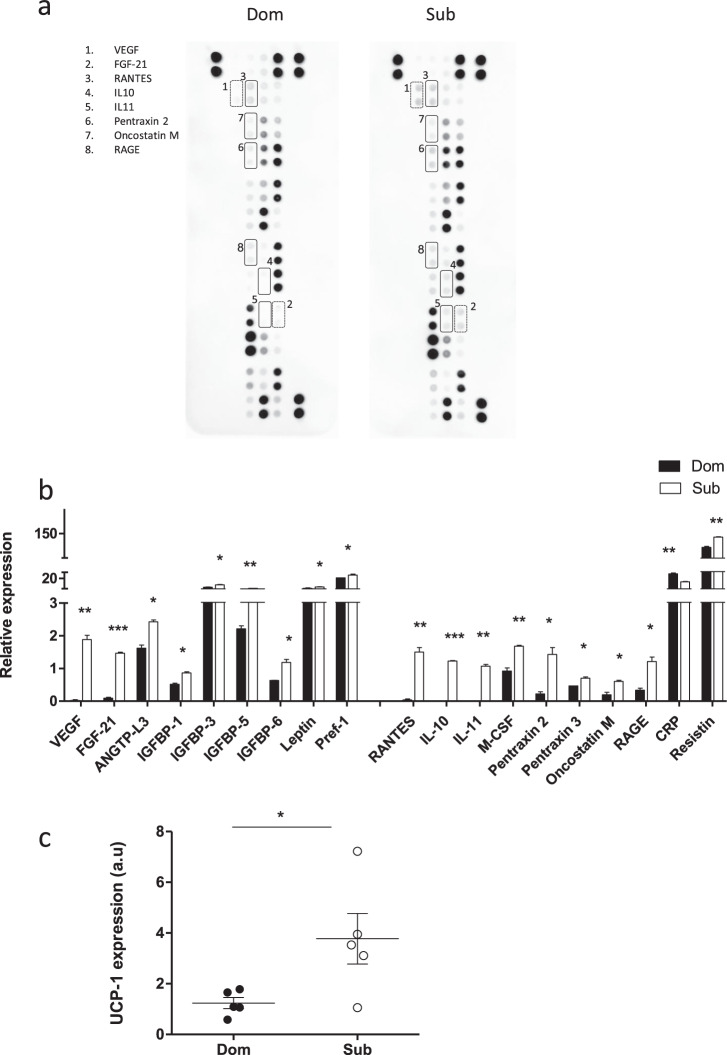
Adipokine profile demonstrates elevated adipokine levels in the eWAT of Sub mice. **a** Proteins extracted from either Dom or Sub eWAT specimens were compared using an adipokine array. **b** Each duplicate was normalized to the positive control. The differences in protein expression of Dom and Sub mice normalized to the corresponding controls were analyzed. Each bar represents the average of adipokine duplicates on the same membrane measured in a pool of eWAT protein extracts from Dom and Sub mice (*n* = 4 in each pool). Only eWAT adipokines that were significantly different among Dom and Sub mice are presented. **c** UCP-1 (marker for browning) mRNA gene expression is higher in Sub compared to Dom mice (*n* = 5 in each group). HPRT was used as a housekeeping gene. a.u: arbitrary units. *p < 0.05, **p < 0.01, ***p < 0.001 analyzed by Student’s *t* test. Error bars show standard deviation.

To further explore the differences between Dom and Sub mice, we analyzed the eWAT adipokine profile (Fig. 3). A comparative analysis revealed that the levels of 18 out of 38 tested adipokines, related to adipogenesis, metabolism homeostasis, and inflammation, were significantly elevated in Sub mice, as compared with their Dom counterparts (*p* < 0.05; Fig. 3b). In contrast, only a single adipokine, C-reactive protein, was significantly elevated in Dom mice, as compared with Sub mice. Sub mice also showed a significant increase in the gene expression of uncoupling protein 1 (UCP-1), a recognized marker of adipose tissue thermogenesis and browning, suggesting differences in energy expenditure between Dom and Sub mice (Fig. 3c).

### Sub and Dom mice possess distinct gut microbiota compositions

**Fig. 4:**
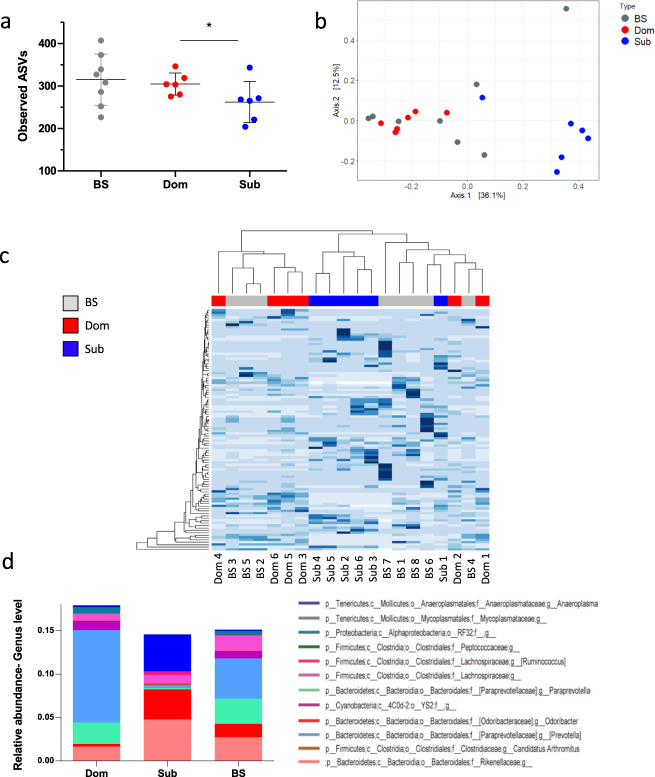

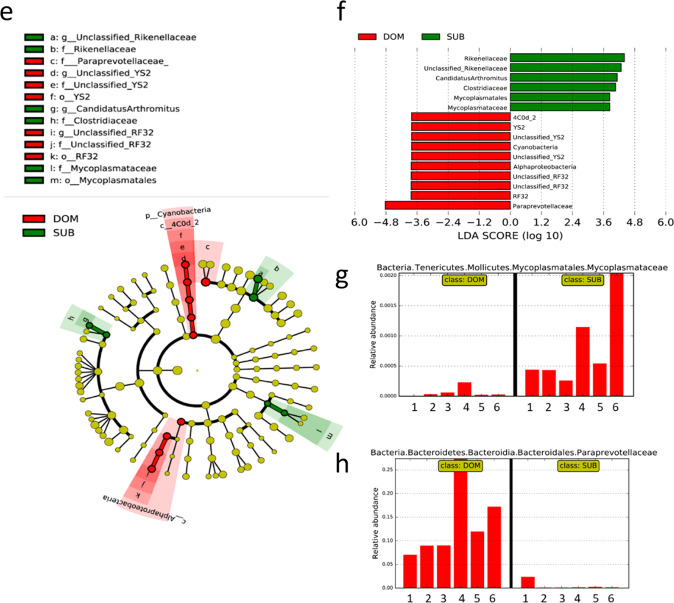
Gut microbiota compositions and unique taxa in Dom, Sub, and BS mice. **a** Alpha diversity of the gut microbiome of Dom, Sub, and background-strain (BS) mice (*n* = 20; SD = 26.10, 48.44, and 60.15, respectively). The alpha diversity of BS and Dom mice was not significantly different. **b** A principal component analysis showing the clustering of the gut microbiome of mice with the same social behavior phenotype. **c** A heatmap of the 100 most variant species identified; taxa with similar distributions are grouped together. **d** Relative abundance up to the genus level. **e** Cladogram of gut microbiota in the different mouse groups. The taxonomic levels are represented by rings, with the class in the outermost ring and the phylum in the innermost ring. Each ring represents a member within that level. Dom and Sub mice are colored in red and green, respectively. **f** The LDA scores of biomarkers found by LEfSe to be significantly different between Dom and Sub mice. Each bar represents the log10 effect size for each taxon. **g**, **h** Abundance histograms of the Mycoplasmataceae (**g**) and Paraprevotellaceae (**h**) biomarkers detected by LEfSe. Each bar represents the relative abundance of the specified taxa in an individual mouse. Statistical significance was assessed by using a one-way ANOVA with Bonferroni correction, *p < 0.05. Error bars show standard deviation.

The pronounced lower body weight and eWAT mass of Sub mice, as compared with Dom mice, has led us to hypothesize that the gut microbiome composition is different in the two groups, which may have altered their energy homeostasis and inflammatory levels. To test this hypothesis, we performed a 16S rRNA sequencing to determine the gut microbiota composition of Dom, Sub, and the parental background strain (BS). This analysis revealed significant differences in the taxonomic composition between Dom and Sub mice, such that individual mice that demonstrated the same behavioral phenotype also demonstrated similar gut microbiota compositions (Fig. 4c). Specifically, the gut microbiome composition of Sub mice was different and less diverse than those of Dom and BS mice (average number of operational taxonomic units (amplicon sequence variants (ASVs)): Sub: 262 ± 48.44 ASVs; Dom: 304.5 ± 26.1 ASVs; BS: 314.8 ± 60.15 ASVs; Fig. 4a). The degree of microbial phylogenetic similarity of BS, Dom, and Sub mice, measured as the unweighted beta diversity, was significantly different between the groups (BS vs. Sub, *p* = 0.007; BS vs. Dom, *p* = 0.07; of Dom vs. Sub, *p* = 0.002; Fig. 4b). Other measures of alpha and beta diversity, generated in QIIME2, provided similar results (in Supplementary Fig. 1 and 2).

Another significant difference between the gut microbiota of Dom and Sub mice was observed in the relative bacterial abundances in each group. A linear discriminant analysis (LDA) of the effect size (LEfSe) indicated that the gut microbiome of Sub mice demonstrated a higher abundance of the Mycoplasmataceae family, the genus *Anaeroplasma* of the Tenericutes phylum (relative abundance: 0.042 ± 0.046), and the Rikenellaceae (0.0478 ± 0.0147) and Clostridiaceae (0.0012 ± 0.0006) families, as compared with Dom mice (0.0021 ± 0.0044, 0.0165 ± 0.0055, and 0.0001 ± 0.0004, respectively) and BS mice (0.0016 ± 0.0026, 0.0273 ± 0.0172, and 0.000442 ± 0.000675, respectively). However, at the family level, the Paraprevotellaceae family (*Paraprevotella* and *Prevotella* at the genus level) was significantly more abundant in the Dom and BS microbiota (0.1313 ± 0.07479 and 0.07543 ± 0.07863, respectively) than in the Sub microbiota (0.004851 ± 0.008412; Fig. 4d, g, h).

### FMT from Sub mice confers Sub behavioral patterns and Sub eWAT features in GF mice

**Fig. 5:**
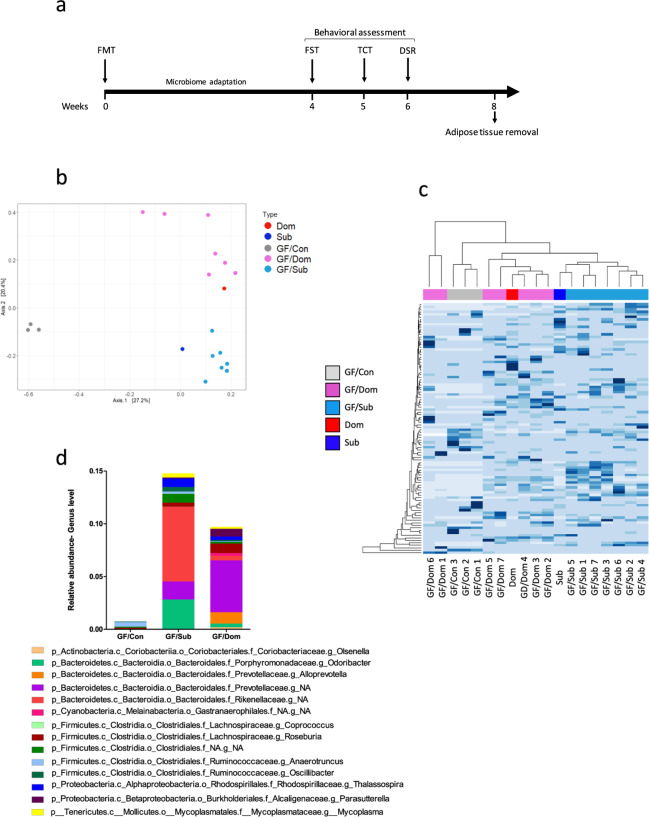

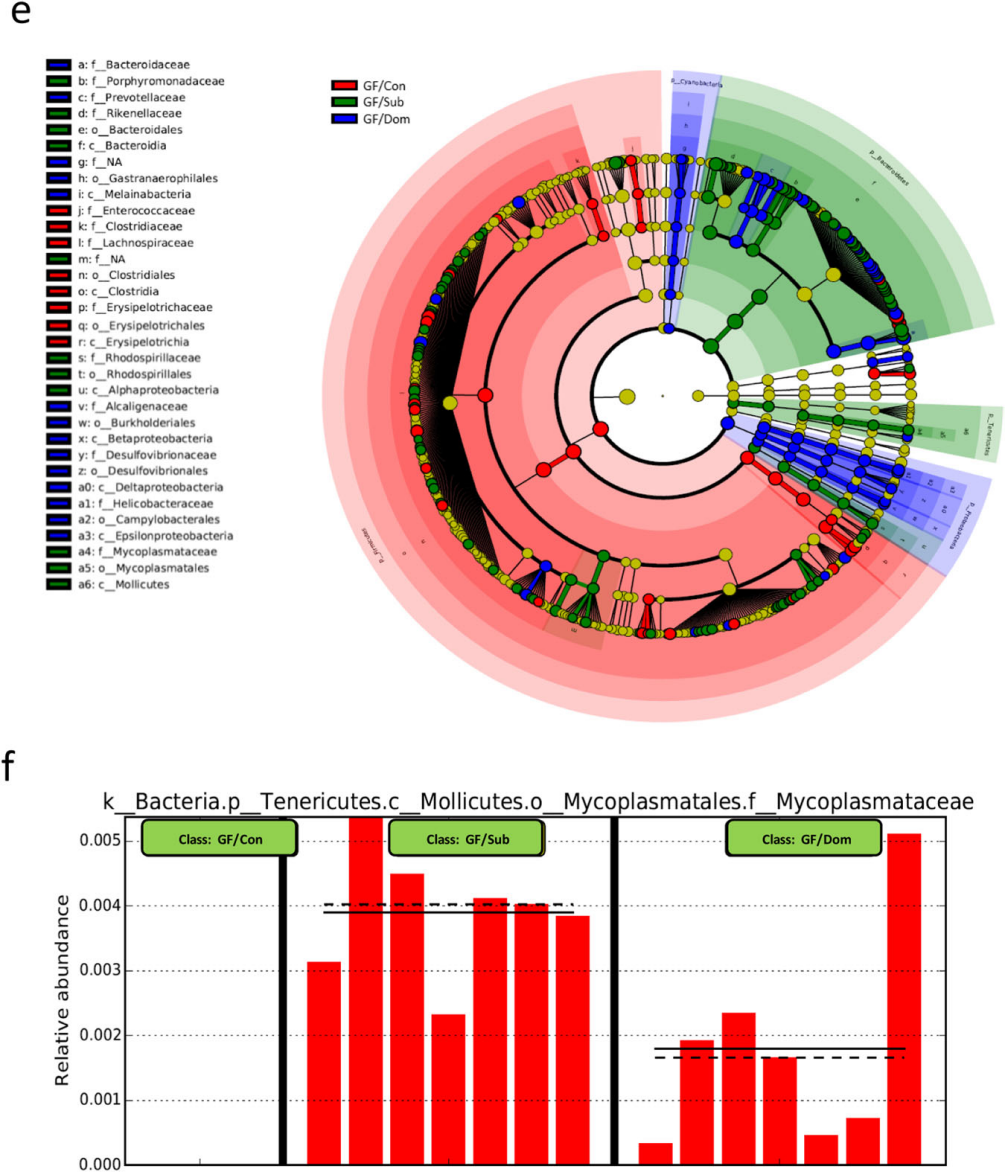
Gut microbiome composition of germ-free mice after a fecal microbiota transplantation from Dom or Sub donor mice. **a** Experimental timeline. Germ-free (GF) mice (*n* = 17) underwent a fecal microbiota transplantation (FMT) of either Dom-derived microbiota (GF/Dom, *n* = 7), Sub-derived microbiota (GF/Sub, *n* = 7), or PBS (GF/Con, *n* = 3). After an adaptation period, the mice underwent a series of behavioral assessments (FST; forced swim test; TCST: three-chamber Sociability test; DSR: dominant–submissive relationship test) and their adipose tissues were removed and analyzed. **b** Principal component analysis of the gut microbiota of GF/Con, GF/Dom, and GF/Sub mice, (*n* = 17) at Day 7 post-FMT, demonstrating the clustering of the gut microbiome in each group, as well as in GF mice and in the donor mice. **c** A heatmap of the microbial composition of the samples at the species level, with the top 100 most variant species identified. Taxa with similar distributions are grouped together. **d** Relative abundance up to the genus level. An additional figure up to the order level (for significantly altered bacteria) is presented in Supplementary Fig. 4. **e** LEfSe cladogram of gut microbiota in the different mouse groups. Taxa whose distributions among different groups are significantly different (*p* < 0.05 and the effect size >2). **f** Abundance histograms of the Mycoplasmataceae biomarker in Sub mice, detected by LEfSe as the marker of GF/Sub mice. Each bar represents the relative abundance of the specified taxa in an individual mouse. Statistical significance was assessed using a one-way ANOVA with Bonferroni correction.

To elucidate the influence of gut microbiome on behavior and on metabolic and inflammatory profiles, we performed an FMT from Dom and Sub mice to Swiss Webster GF mice. First, we confirmed the establishment of the Dom- or Sub-derived bacterial communities in the GF-transplanted mice. To this end, we used 16S rRNA gene sequencing of fecal samples collected 7 days post-FMT from the Dom-transplanted GF mice (GF/Dom), Sub-transplanted GF mice (GF/Sub), and from the Dom or Sub donor mice. There were no differences in alpha diversity between GF/Dom and GF/Sub mice. The composition of the gut microbiome was similar in all transplanted GF mice within each group, and it resembled the composition in the donor mice (Fig. 5b, c). Other measures of alpha and beta diversity, which were generated in QIIME2, provided similar results (in Supplementary Fig. 1 and 2). An indication that the GF/Sub mice acquired the Sub-derived microbiota was the significant increase in the abundance of the genus *Mycoplasma* and the Rikenellaceae family (Fig. 5d–f), identified as a Sub mouse gut microbiome biomarker by the LEfSe analysis (Fig. 4g).

**Fig. 6:**
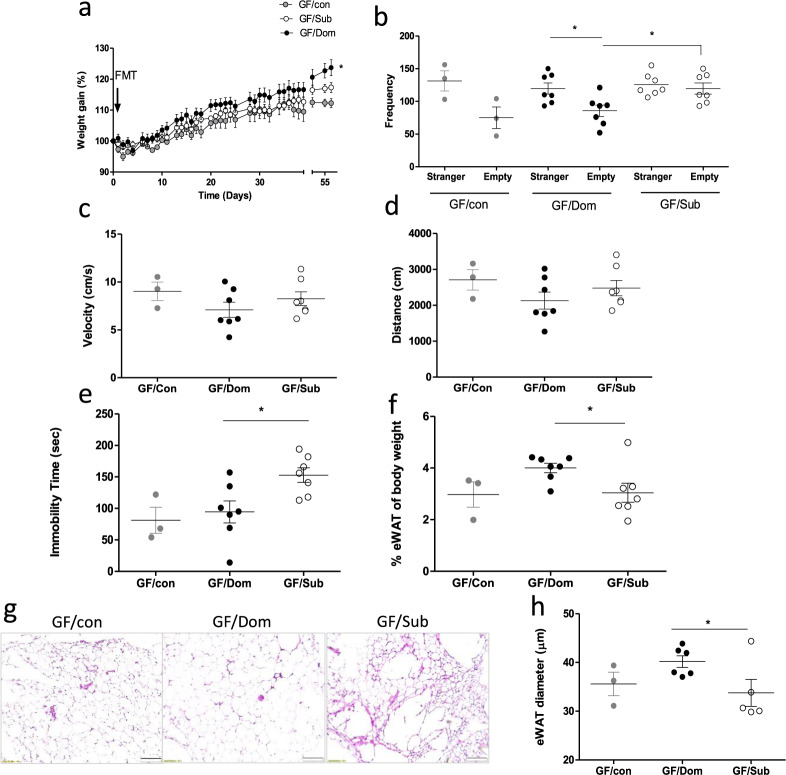

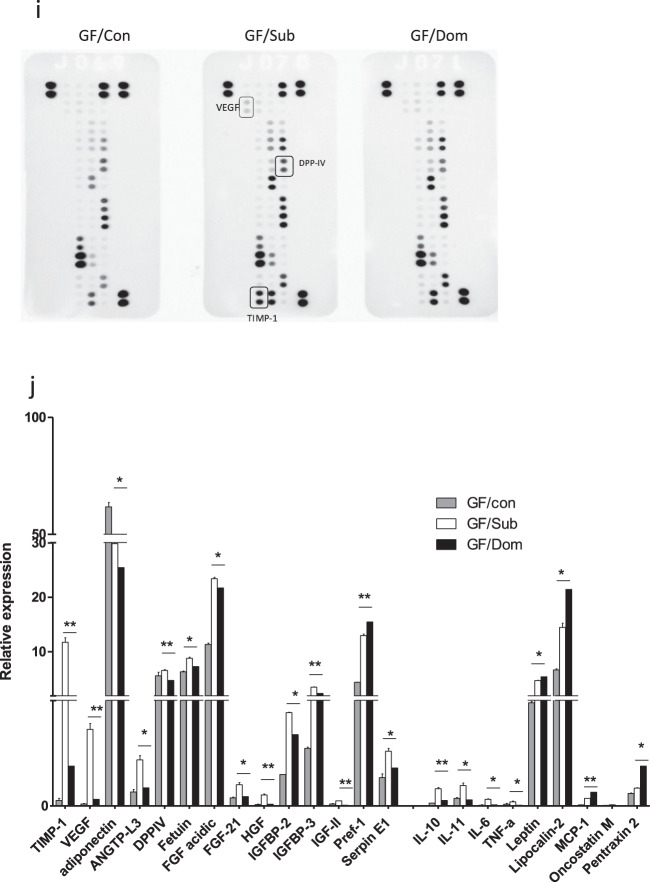
The effects of FMT on mouse body weight, behavior, eWAT content, and adipokine profile. **a** Body weight follow-up of transplanted GF mice. The curves represent the average mouse weight gain as a percentage from their weight at the day of transplantation. **b**–**d** Mouse social behavior, measured in the TCST. **c**, **d** The average velocity (**c**) and distance (**d**) per group shows similar locomotor activity in all the groups. **e** Depressive-like behavior, reflected in immobility time in the FST, in GF/Sub mice, as compared with normal behavior in GF/Dom mice. **f** Quantification of eWAT mass, obtained from GF mice, normalized to the respective body weight of each mouse. **g** Histological (H&E) staining of eWAT from GF-transplanted mice (×40 magnification, scale bar = 20 μm). **h** The eWAT cell diameter of transplanted GF mice was determined using the ImageJ software. Ten random cells per field were analyzed from 10 random fields in each mouse. **i** An adipokine array comparison of pooled proteins extracted from the eWAT of GF/Sub (*n* = 4) and GF/Dom (*n* = 4). **j** The eWAT adipokines that their expression was significantly altered following FMT. Each bar represents the average of duplicate adipokine expression normalized to the positive control. GF/Sub mice demonstrated a significantly higher adipokine level than GF/Dom mice. Statistical significance was determined using Student’s *t* test or one-/two-way ANOVA with Bonferroni correction, *p < 0.05, **p < 0.01. Error bars show standard deviation.

A single Sub-derived FMT in GF mice resulted in weight gain in all the transplanted GF mice, while GF/Dom mice gained more weight than GF/Sub mice 55 days after inoculation (Fig. 6a). In addition, FMT resulted in significant behavioral changes, which were observed 4 weeks post-transplantation. Specifically, GF/Sub mice adopted the naive submissive behavioral characteristics of their donor Sub mice, namely, anti-social behavior measured in the three-chamber sociability test (TCST)^30^ and depressive-like behavior reflected in the forced swim test (FST)^32^. In the TCST, GF/Sub mice showed a similar entrance frequency to the empty room and to the room with the stranger mouse (Fig. 6b, *p* = 0.0157), and these changes did not result from altered velocity or locomotor disabilities. In contrast, GF/Dom mice significantly preferred to enter the room with the stranger mouse (Fig. 6c, d). In the FST, GF/Sub mice showed increased immobility time (*p* = 0.0068), as compared with GF/Dom mice (Fig. 6e). Additional behavioral tests like the elevated plus maze^37^ and the open field^38^ were performed on all transplanted GF mice without apparent differences between groups; for example, in the DSR test, the last-day results were 26 and 60 s for the GF/Dom and GF/Sub mice, respectively (*p* > 0.05; in Supplementary Fig. 3).

The observed behavioral changes significantly correlated with changes in adipose tissue mass, cell size, and the adipokine profile. The eWAT mass (normalized to mouse weight) was markedly lower in GF/Sub mice than in GF/Dom mice (30 ± 3% vs. 40 ± 1%, respectively; *p* = 0.0371; Fig. 6g) and the adipokine profile of GF/Sub and GF/Dom mice was similar to that of their respective donor mice (Fig. 6i, j).

## Discussion

We show herein that adult mice from closely related genetic backgrounds but exhibiting a distinct social characteristic of dominance or submissiveness possess a different gut microbiota composition, which corresponds to their behavioral features. The distinct gut microbiome of each mouse subpopulation may explain the body weight differences between Dom and Sub mice, which were observed from 2 weeks of age and persisted throughout maturation^39^. An FMT of Sub microbiota into GF mice resulted in significant behavioral changes, including an impairment in social skills and an increase in depressive-like symptoms, which are typical of Sub mice behavior. Physiological changes in these mice included reduced eWAT mass, smaller adipocytes, and an altered inflammatory outline—adopting the major eWAT features of Sub mice. Overall, we conclude that Sub microbiome-transplanted GF mice acquired the major behavioral and physiological characteristics of Sub mice.

Gut microbiome analyses pointed out that the Dom mice gut microbiome was relatively similar to that of the BS, while the Sub mice microbiome was unique with reduced alpha diversity and with specific bacterial families of Mycoplasmaceae, Rikenellaceae, and Clostridiaceae, lacking the Paraprevotellaceae family, which was presented only in the Dom and BS mice. It is important to underline that, similarly to our findings, a reduction in gut microbiome richness and diversity was also observed in C57BL/6J mice following social defeat and in mice exposed to chronic mild stress^40^. In humans, lower gut microbiota diversity was reported in individuals with psychiatric disorders, including depression^41,42^ and anxiety^42,43^. Notably, in humans, subordinate behavior has been proposed as an important element in the etiopathology of depressive disorders^31^, thus the low bacterial diversity that we observed in the gut microbiome of Sub mice is in agreement with observations in humans with major depressive disorder (MDD)^41^.

An important finding in this study was the unique presence of specific bacterial genera*—Mycoplasma* and *Anaeroplasma* of the Tenericutes phyla (Mycoplasmataceae and Anaeroplasmataceae)—in the Sub gut microbiome and the link between these genera and submissive behavior. These results are inconsistent with those of a previous study, which reported a decrease in the abundance of Tenericutes in stress-sensitive C57BL/6 mice after chronic social defeat^42^, and study which reported a higher abundance of Tenericutes levels in rats that demonstrated increased social interactions after providing them access to high-fat-high-sugar food^44^.

Another unique biomarker that we found in stress-sensitive Sub mice was the bacterial family Rikenellaceae, which was reported in other mouse studies to be correlated with stress and eWAT inflammation. For instance, this bacterial family was reported to be abundant in C57BL/6J male mice that had been exposed to 12 weeks of continuous dark stress^45^ and in adult B6129SF2/J mice that were born to high-fat-diet dams and suffered from memory and exploratory behavior dysfunctions^46^. We did not find any support in the literature for a correlation between Clostridiaceae and behavioral abnormalities.

The reduced abundance of the *Paraprevotella* and *Prevotella* genera in the Sub mice microbiota could also be associated with the vulnerability of these mice to stress. Similar findings were described in mice that had been exposed to chronic mild social defeat stress^24^, while a decrease in *Prevotella* abundance has also been noted in a study of humans with MDD^47^.

The involvement of the gut microbiome in shaping behavioral patterns was supported by the FMT experiment, in which GF mice, upon a single transplantation, adopted the Sub behavioral patterns, including the asocial (TCST) and depressive-like (FST) behaviors. Interestingly, when we tested GF/Dom vs. GF/Sub mice in the DSR test, the transplanted GF mice did not show apparent DSR patterns. This finding may be explained by the fact that we performed only a single transplantation, and, perhaps, additional transplantations would have conferred a more pronounced effect on the DSR paradigm. Moreover, while Sub mice possessed lower alpha diversity compared to the Dom mice, the observed ASVs of the transplanted GF mice in both GF/Dom and GF/Sub did not differ significantly. We may explain this by the fact that FMT was carried out with a pool of feces (collected from four mice with the respective behavioral phenotype) and not with a single mouse feces or due to exposure to aerobic conditions, which might influence the viability of some bacteria. Moreover, we performed only a single FMT and it is reasonable to expect that additional transplantations would increase the significant difference between the mice. Furthermore, although we expect to find a lower diversity in the GF/Sub mice gut microbiome, these mice acquired the Sub gut microbiome pattern.

Considerably, alongside the acquisition of the Sub behavioral patterns, transplantation was accompanied by the adaptation of the Sub eWAT metabolic and inflammatory features. Whether and how eWAT metabolism and inflammation directly influence the social behavior mode can only be speculated at this point; the importance of eWAT metabolic and inflammatory adipokines to brain health has been previously discussed^48^. Chronic social defeat stressed C57BL/6J mice demonstrated a decreased expression of PPARγ, a key transcriptional factor that controls adipokine gene expression, suggesting a correlation between eWAT physiology, social interactions, and depression^49^. Excessive adipose expansion during obesity causes adipose tissue dysfunction and increase in proinflammatory factors, consequently leading to systemic inflammation^50^. In another study, NLRP3 inflammasome knock-out mice exhibited a reduced depressive-like behavior following chronic stress, alongside increased *Prevotella* levels, thus linking inflammation, pathologic behavior, and microbiome composition^51^.

Numerous human studies focus on the association between inflammation and MDD^52^. Indeed, inflammation and the release of inflammatory cytokines have been shown to affect the brain circuitry of individuals with MDD, which contributed to their behavioral changes^53^. In another study that correlated adipose inflammation and depression, symptoms of depression were shown to promote weight accumulation, which in turn activated an inflammatory response of IL-6 and Leptin^54^. In contrast, while Sub mice show a depressive-like behavior and a pronounced elevation in eWAT metabolic and inflammatory markers, their microbiome promoted a lean, rather than obese, phenotype. The lean Sub mice phenotype is in agreement with a study that described an association between mood disorders and high expression of genes related to adipose tissue inflammation in non-obese rather than in obese patients^55^.

Numerous adipokines were significantly upregulated in both Sub and GF/Sub compare to Dom and GF/Dom mice. The most pronounced differences were observed in fibroblast growth factor 21 (FGF-21) and vascular endothelial growth factor (VEGF). FGF21 is a key regulator of adipocyte browning and acts through modulation of UCP-1 expression^56^, which correlates with the higher UCP-1 mRNA expression we observed in Sub mice. VEGF is critical for adipose macrophage infiltration and its effect is mediated by VEGFR-3, which is upregulated on pro-inflammatory M1 polarized macrophages^57^. Thus induction of these adipokines in both Sub and in FMT GF/Sub mice further supports our speculation that adipose inflammation is triggered by factors derived from gut microbiota.

In conclusion, our study provides new insights into social interaction abnormalities and reveals a direct link between inherited stress vulnerability, the gut microbiome composition, behavioral patterns, and adipose tissue metabolism and inflammation. A single FMT of Sub microbiota into GF mice led to a reduced body weight and was correlated with a significant decrease in eWAT mass, accompanied by induced inflammation and metabolic alterations. The fact that Sub-transplanted GF mice acquired the entire set of Sub features confirms the strong link between gut microbiota composition, adipose tissue physiology, and social behavior.

## Materials and methods

### Animals

All animal experiment protocols were reviewed and approved by the Ariel University Institutional Ethical Committee (Protocols IL-122-01-17, IL-178-03-19). Dom and Sub male and female mice were developed by selective breeding, as described elsewhere^29^. Descendants of generations F24–F26 were used in this study. The Sabra outbred strain (ENVIGO, Rehovot, Israel) served as a parent generation for the selective breeding and was used as a BS in this study^58^. Mice belonging to different behavioral phenotypes (Dom, Sub, or BS) were housed in separate cages. All mice were maintained under 12-h light/dark cycle conditions in a controlled atmosphere at 24 °C. During all experiments, mice had free access to chow and water.

### DSR test

Dom and Sub mice used in this study were selectively bred on basis of their behavior in the DSR test. The DSR test is a food competition paradigm used to assess social interaction between pair of mice as described previously^29,34^. Briefly, pairs of 8-week-old mice from the same sex but from different home cages were matched for relatively similar weights (average 43.7 ± 2.1 g) and were tested according to the DSR protocol in the DSR apparatus. Made up from Plexiglas, the DSR apparatus consisted of two identical chambers (12 cm × 8.5 cm × 7 cm) placed on opposite sides and connected by a tunnel (2.5 cm × 2.5 cm × 27 cm). In the center of the tunnel, a feeder tube with a 0.5 cm diameter hole in its bottom provides a sweetened milk (3% fat, 10% sugar), to which only one animal has access at any given time. On the tunnel, at the entrance to each chamber, a gate prevents mice from reaching the milk until it is removed, to create an equal starting point at the beginning of each session. Fourteen hours before each session, the mice were deprived of food, and water was provided ad libitum. On the day of the test, a pair of mice were placed in the two separate chambers behind the gates. Mice were left in the chamber for 30 s for habituation. The gates were then removed, and during 5 min the milk-drinking time was recorded manually for each mouse. DSR sessions were carried out for 4 consecutive days. A DSR was determined if a significant difference (*p* < 0.05) was observed between the average daily drinking durations of each mouse in a pair and if the difference in drinking scores was at least 40%. According to these criteria, >99% of selectively bred dominant and submissive mice developed strong and stable DSRs. A detailed description of the DSR procedure, selection criteria, and DSR apparatus scheme was published elsewhere^29^.

### Three-chamber sociability test

We employed the TCST to assess the motivation of the fecal microbiota transplanted GF male mice to socially interact with a stranger mouse. A mouse was placed in between three separated chambers, with free entry to all chambers. On one of the two side chambers, a mouse of the same strain as the mouse being tested but that had had no previous contact with the tested mouse was caged inside a cylinder. An empty cage was placed on the other side of the chamber. The number of entries of the tested mouse to each side of the chamber was measured for 10 min. Mouse movements were recorded using EthoVision 9.1 (Noldus, the Netherlands)^13^.

### Forced swim test

To evaluate depressive-like behavior, in the GF-transplanted male mice we measured the immobility time of mice placed for 6 min in a 5-L water-filled cylinder (25 cm height, 10 cm water depth, water temperature: 25 ± 1 °C). The total duration of immobility was recorded during the last 4 min of the trial, allowing the first 2 min for adjustment. The duration of immobility was defined as the time during which the mouse remained immobile, made no attempts to escape, and showed only slow movements to keep its head above the water^59^.

### Fecal sample collection and DNA purification

Fecal samples were collected from 20 naive 10-week-old Sabra (BS, males *n* = 4 and females *n* = 4) Dom (males *n* = 6 and females *n* = 1), and Sub (males *n* = 5 and females *n* = 1) mice and from 17 male GF-transplanted mice. Fresh fecal samples (~250 mg each) were collected from 9:00 a.m. to 12:00 a.m. from each mouse, which were placed individually in a sterile cage during fecal collection. The fecal samples were collected using sterile forceps, placed in pre-weighed sterile Eppendorf tubes, and immediately stored at −80 °C. All fecal samples were collected prior to the DSR session to ensure that the gut microbiota results are not influenced by differences in milk consumption or stress.

Genomic DNA was isolated from fecal samples using the QIAamp DNA Stool Mini Kit (Qiagen, Hilden, Germany) according to the manufacturer’s instructions, with an additional homogenization step performed for each isolation using a Pestle Motor Mixer in an RNase/DNase/Pyrogen-free Microtube (Argos Technologies, Inc. Vernon Hills, IL) to optimize the DNA yield. DNA concentration and purity were quantified (Nanodrop 2000 Thermo Fisher Scientific, Waltham, MA), followed by gel electrophoresis (1.0% agarose). The purified DNA samples were stored at −20 °C for further analysis.

### PCR and sequencing

The 16S rRNA gene was amplified by using the Takara PrimeSTAR Max DNA Polymerase with the V4 (515F barcoded-806R) primer set 16S_515FBC_ILA: AATGATACGGCGACCACCGAGATCTACACGCTAGCCTTCGTCGCTATGGTAATTGTGTGYCAGCMGCCGCGGTAA;16S_806RNBC_ILA:CAAGCAGAAGACGGCATACGAGATAGTCAGTCAGCCGGACTACHVGGGTWTCTAAT.

#### PCR preparation

2×PrimeSTAR Max (Takara-Clontech, Shiga, Japan) Readymix 25 µl, 515F primer (10 µM) 2 µl, 806 R primer (10 µM) 2 µl, DDW 17 µl, DNA template 4 µl. PCR conditions: 95 °C 180 s; 30 cycles of [98 °C 10 s, 55 °C 5 s, 72 °C 20 s]; 72 °C 60 s; 4 °C 10 min; 10 °C hold. A stool sample was included as a positive control and ultra-pure water as a negative control. Products were run on a 1.5% agarose gel for imaging. Amplicons were then purified by using Agencourt AMPure XP magnetic beads (Beckman Coulter, Brea, CA) and subsequently quantified using the Quant-It Picogreen dsDNA Quantitation Kit (Invitrogen, Carlsbad, CA). Equimolar amounts of DNA from individual samples were pooled and sequenced using the Illumina MiSeq platform at the Genomic Center of Bar-Ilan University, Azrieli Faculty of Medicine, Safed, Israel.

### Taxonomic analysis of BS, Dom, and Sub mice

FASTQ data were processed and analyzed by using QIIME2 pipeline, version 2019.4^60^. Single-end sequences were first demultiplexed using the q2‐demux plugin. To improve taxonomic resolution, reads were denoised and clustered using DADA2 via q2‐dada2^61^. MAFFT^62^ and fasttree2^63^ were used for alignment and phylogeny construction for all ASVs, using q2‐alignment and q2‐phylogeny plugins, respectively. Taxonomy classification was accomplished by using a q2‐feature‐classifier^64^, while final feature sequences were aligned against the Greengenes database with 99% confidence^65^. To avoid any possible contamination, the feature table was filtered via a q2-feature-table. First, features that were annotated as mitochondria or chloroplast were filtered out. Next, features that were found in ≤10% of the samples from the total number of samples were removed, and features with <0.001% frequencies in total were removed.

The analysis was performed on a rarefied table of 76,000 reads per sample. Alpha diversity was calculated using the Faith’s Phylogenetic Diversity^66^ measure, referring to bacterial richness within the sample, while significant differences in bacterial richness between the groups were tested using Kruskal–Wallis test. Beta diversity was analyzed according to weighted (quantitative)^67^ and unweighted (qualitative)^68^ UniFrac distances in order to compare differences in gut bacterial communities between the sample groups. To evaluate the level of significance, a permutational multivariate analysis of variance was performed, as implanted in QIIME2 with the default of 999 permutations, both weighted and unweighted UniFrac.

A principal component analysis (PCA) and heatmap plots were generated using the “R” (https://www.r-project.org/) software (version 3.6.3). Beta diversity was assessed using a Bray–Curtis dissimilarity calculator (a commonly used Beta diversity index) and calculated using the ordinate() function in the phyloseq package 1.30.0^69^. The distance matrices were visualized by using PCA. Taxonomy relative abundance was generated from the QIIME2 software. A heatmap was generated by calculating the standard deviation (SD) of the relative abundance of each taxon and plotting the 100 most dissimilar SDs in a heatmap.

Significant differences in bacterial genus-level abundance between the Dom and Sub groups were determined using a LEfSe with an LDA score >2.0 and *α* values of 0.05 ^70^.

### Mouse body weight follow-up, food intake measurements, and adipose tissue removal

Mouse body weight follow-up was determined for naive male and female Dom and Sub mice during an 8-week period from birth to adulthood. Forty-four mice from each group were divided into eight cages and chow was weighed on a weekly basis, beginning at 4 weeks of age, both before and after filling. To determine the weekly chow intake, the amount of chow consumed per cage was divided by the average body weight of the caged mice. Adipose tissue analysis was performed on male adult (3 months old) mice. eWATs were dissected aseptically from each mouse (*n* = 10 mice in each group), weighed, and divided for further analysis, including histology and quantitative reverse transcription PCR (q-RT-PCR).

### Histology

eWAT specimens were collected from *n* = 10 Dom and *n* = 10 Sub male mice and incubated in 4% paraformaldehyde for 24 h, after which the specimens were transferred to a 70% ethanol solution. The tissue was embedded in paraffin and sectioned into 4-μm sections. Slides were stained in H&E and immunohistochemistry staining was performed for F4/80 (Abcam, Cambridge, UK) using DAB as the substrate (Zytomed, Berlin, Germany).

### Adipokine array

To screen for differences in adipose tissue between the male Dom, Sub, and GF-transplanted mice, we used an antibody-based protein array [Proteome Profiler: Mouse adipokine array (R&D Systems, Minneapolis, MN)], according to the manufacturer’s instructions. We used a pooled protein mix of four random mice from each group. The average signal of pixel density from duplicate adipokines was determined using the ImageQuant TL software. The relative intensity of the reference values (three inside control duplicates in each membrane) was included in the densitometry calculations.

### Fecal transplantation experiment

Male GF Swiss Webster mice were inbred and housed in semi-solid GF isolators (3–5 mice per cage) at the Azrieli Faculty of Medicine (Safed, Israel) under a 12-h light/12-h dark cycle, at 22 °C, with autoclaved food and water available ad libitum. Fecal transplantations were performed in 8–10-week-old GF mice (*n* = 17). Fresh fecal samples were collected and pooled for transplantation from adult (12 weeks) donor Dom (*n* = 4) or Sub (*n* = 4) mice. These mice were housed in individual cages together with four other mice (Dom or Sub) that were not used for this experiment (In our mice facility, we house 5 mice per cage.). The stools that were collected from 4 mice (per each behavioral phenotype) were pooled into one tube that served as the pooled fecal material for transplantation to each GF group (GF/Dom and GF/Sub). After the FMT experiment, we housed the transplanted GF mice as follows (GF/Dom: 4 and 3 per cage and GF/Sub: 4 and 3 per cage). Mouse inoculation of the respective fecal material (Dom/Sub/CON, 200 µl) was performed by oral gavage using a sterile feeding tube (20 ga × 38 mm, Instech Laboratories, Inc., Plymouth Meeting, PA) after suspending each stool pellet in 1 ml of sterile phosphate-buffered saline. The effect of fecal transplantation on GF mouse behavior was assessed using the TCST and FST, which were performed 28–35 days post-transplantation.

### Gut microbiome analysis of GF-transplanted mice

The gut microbiome of GF-transplanted male mice (*n* = 17) was analyzed by ZymoBIOMICS® Targeted Sequencing Service for Microbiome Analysis (Zymo Research, Irvine, CA). The ZymoBIOMICS®-96 MagBead DNA Kit was used to extract DNA, using an automated platform. Bacterial 16S ribosomal RNA gene-targeted sequencing was performed by using the Quick-16S™ NGS Library Prep Kit (Zymo Research). The bacterial 16S primers amplified the V3–V4 region of the 16S rRNA gene. The sequencing library was prepared by using an innovative process in which PCR reactions were performed in RT-PCR machines to control cycles and, therefore, limit PCR chimera formation. The final PCR products were quantified by qPCR fluorescence readings and pooled together based on equal molarity. The final pooled library was cleaned with the Select-a-Size DNA Clean & Concentrator™ (Zymo Research) and then quantified with TapeStation® (Agilent Technologies, Santa Clara, CA) and Qubit® (Thermo Fisher Scientific). The final library was sequenced on Illumina® MiSeq™ with a v3 Reagent Kit (600 cycles). The sequencing was performed with a 10% PhiX spike-in.

### Gene expression quantification

RNA was extracted from the adipose tissues of random Dom and Sub male mice (*n* = 5 from each group) using a QIAzol Lysis Reagent and an RNA Extraction Kit (both from Qiagen). Reverse transcription was performed with a commercial cDNA Kit (Promega, Madison, WI). RT-PCR was performed using a SYBR mix (KAPA Biosystems, Wilmington, MA) and the following primers: F4/80, forward 5′-TGACAACCAGACGGCTTGTG-3′ and reverse 5′-GCAGGCGAGGAAAAGATAGTGT-3′; UCP-1, forward 5′-GATGGTGAACCCGACAACTT-3′ and reverse 5′-CTGAAACTCCGGCTGAGAAG-3′; normalized with the housekeeping gene HPRT with the following primers: forward 5′-TTGCGCTCATCTTAGGCTTT-3′ and reverse 5′-TGTTGGATATGCCCTTG-3′ (Hylabs, Rehovot, Israel). Reactions were performed using the AriaMx 96 RT-PCR System (Agilent, Santa Clara, CA).

### Statistical analyses

All statistical analyses were performed using GraphPad Prism 6. Quantitative results are expressed as means ± SEMs and were analyzed using Student’s *t* test for individual comparisons or one- or two-way analysis of variance, followed by Bonferroni means separation test, for multiple comparisons. The statistical significance of differences between groups is presented graphically as * for *p* < 0.05, ** for *p* < 0.01, and *** for *p* < 0.001.

### Reporting summary

Further information on research design is available in the Nature Research Reporting Summary linked to this article.

## Supplementary information

Supplementary

Reporting Summary

## Data Availability

The sequence data have been deposited in the NCBI Sequence Read Archive under accession number PRJNA597453 for BS, Dom, and Sub naive mice and PRJNA635674 for GF-transplanted mice.
