# Supplementary material for: Gut microbiota determines the social behavior of mice and induces metabolic and inflammatory changes in their adipose tissue
Source: NPJ Biofilms Microbiomes. 2021 Mar 19;7:28. doi: 10.1038/s41522-021-00193-9 (PMC7979825; doi:10.1038/s41522-021-00193-9)
Supplement: Supplementary file 1 — Supplementary [file 41522_2021_193_MOESM1_ESM.pdf]

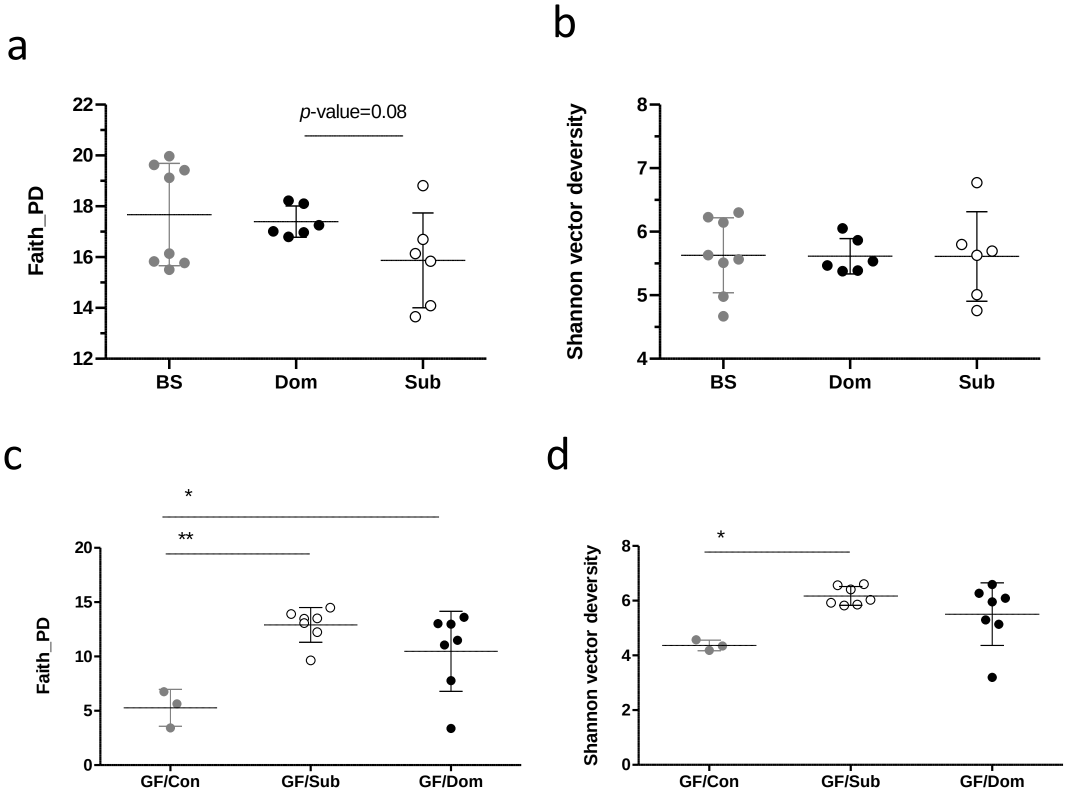

Supplementary Figure 1. Alpha diversity of Dom and Sub mice and swiss webster transplanted mice after FMT. (a) Faith PD analysis of BS, Dom and Sub mice gut microbiome. (b) Shannon vector analysis of BS, Dom and Sub mice gut microbiome. (c) Faith PD analysis of GF/Con, GF/Dom and GF/Sub mice gut microbiome. (d) Shannon vector analysis of BS, Dom and Sub mice gut microbiome. Statistical significance was assessed using a Student's t-test. (*) $p < 0.05$, (**) $p < 0.01$.

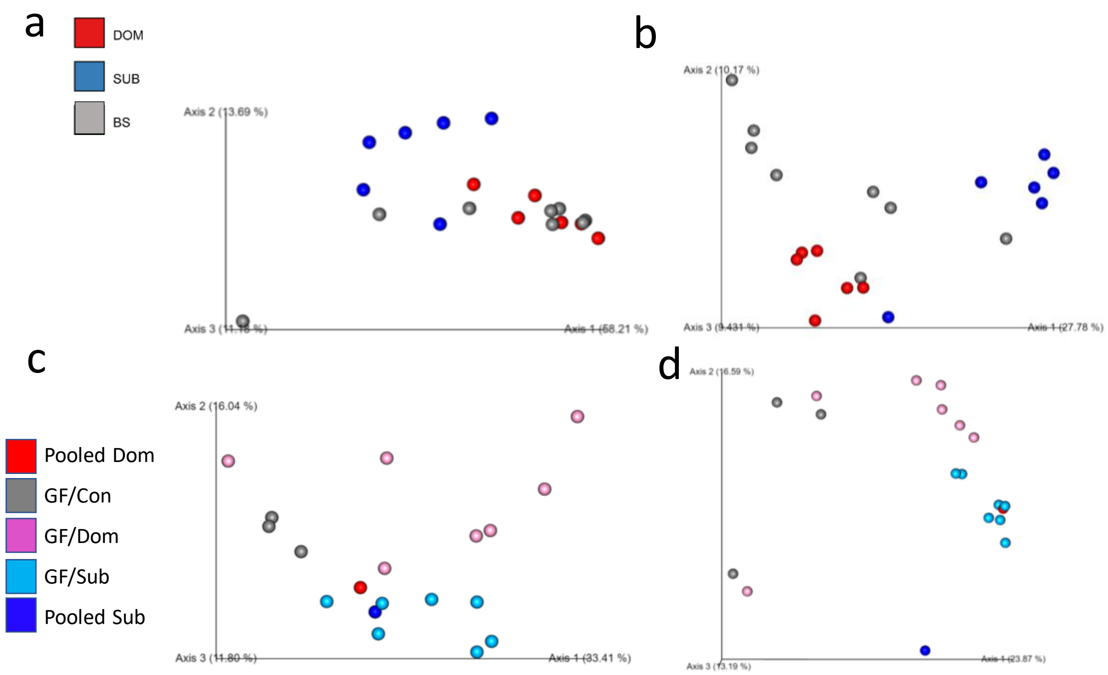

Supplementary Figure 2. Beta diversity of Dom and Sub mice and Swiss Webster GF mice after FMT. (a) weighted analysis of BS, Dom and Sub mice gut microbiome (b) unweighted analysis of BS, Dom and Sub mice gut microbiome. (c) weighted analysis of GF/Con, GF/Dom and GF/Sub mice gut microbiome. (d) unweighted of GF/Con, GF/Dom and GF/Sub mice gut microbiome. All PCA graphs were generated using Qiime2 software.

a

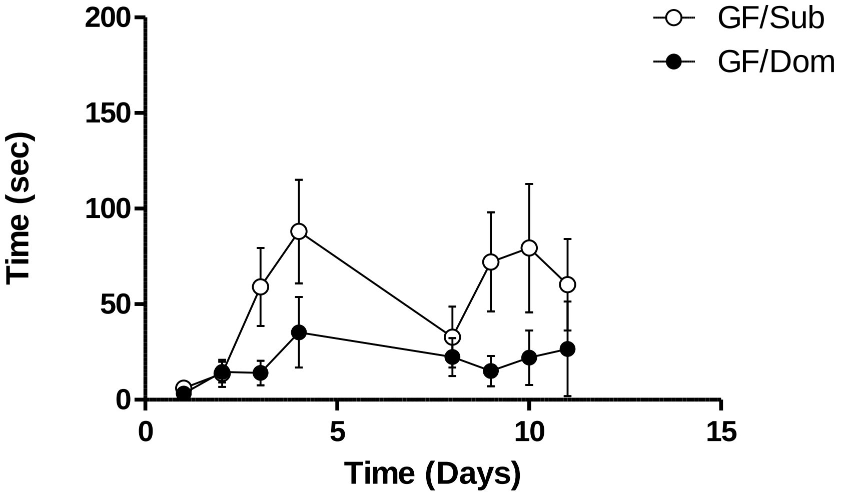

Supplementary Figure 3. Behavioral tests of GF/con GF/Dom and GF/Sub mice. (a) GF/Dom (n=6) and GF/Sub (n=6) mice possess a similar drinking time (p>0.05). Statistical significance was assessed using Two-way ANOVA following a Bonferroni test.

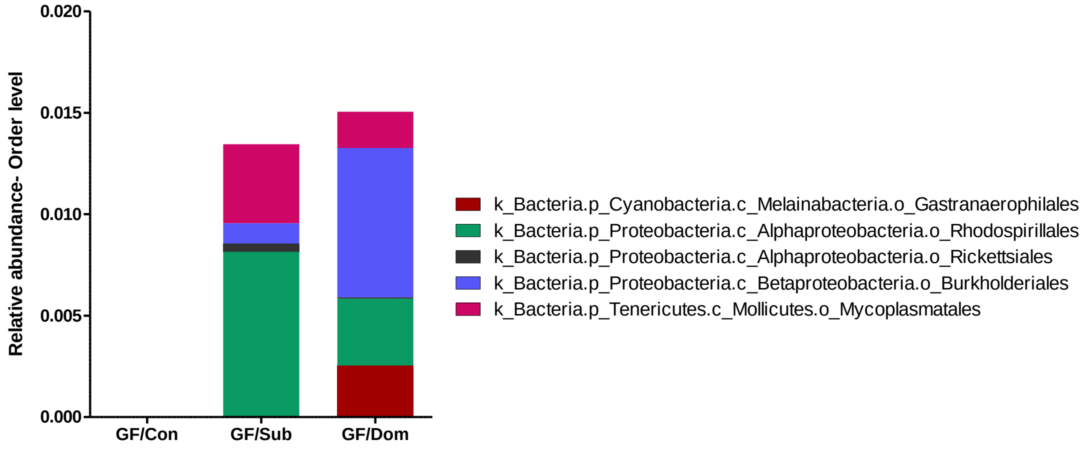

Supplementary Figure 4. Gut microbiome composition of germ-free mice after an FMT from Dom or Sub donor mice. The relative abundance up to the order level. Only the significantly different orders between GF/Dom and GF/Sub mice are presented. Statistical significance was assessed using a student's *t*-test.
