# Supplementary material for: Gut microbiota determines the social behavior of mice and induces metabolic and inflammatory changes in their adipose tissue
Source: NPJ Biofilms Microbiomes. 2021 Mar 19;7:28. doi: 10.1038/s41522-021-00193-9 (PMC7979825; doi:10.1038/s41522-021-00193-9)
Supplement: Supplementary file 2 — Reporting Summary [file 41522_2021_193_MOESM2_ESM.pdf]

nature research

Corresponding author(s): NPJBIOFILMS-00918

Last updated by author(s): Jan 17, 2021

Reporting Summary

Nature Research wishes to improve the reproducibility of the work that we publish. This form provides structure for consistency and transparency in reporting. For further information on Nature Research policies, see our Editorial Policies and the Editorial Policy Checklist.

Statistics

For all statistical analyses, confirm that the following items are present in the figure legend, table legend, main text, or Methods section.

n/a	Confirmed	
☐	☒	The exact sample size (n) for each experimental group/condition, given as a discrete number and unit of measurement
☐	☒	A statement on whether measurements were taken from distinct samples or whether the same sample was measured repeatedly
☐	☒	The statistical test(s) used AND whether they are one- or two-sided<br>*Only common tests should be described solely by name; describe more complex techniques in the Methods section.*
☐	☒	A description of all covariates tested
☒	☐	A description of any assumptions or corrections, such as tests of normality and adjustment for multiple comparisons
☐	☒	A full description of the statistical parameters including central tendency (e.g. means) or other basic estimates (e.g. regression coefficient) AND variation (e.g. standard deviation) or associated estimates of uncertainty (e.g. confidence intervals)
☒	☐	For null hypothesis testing, the test statistic (e.g. F, t, r) with confidence intervals, effect sizes, degrees of freedom and P value noted<br>*Give P values as exact values whenever suitable.*
☒	☐	For Bayesian analysis, information on the choice of priors and Markov chain Monte Carlo settings
☒	☐	For hierarchical and complex designs, identification of the appropriate level for tests and full reporting of outcomes
☒	☐	Estimates of effect sizes (e.g. Cohen's d, Pearson's r), indicating how they were calculated

Our web collection on statistics for biologists contains articles on many of the points above.

Software and code

Policy information about availability of computer code

Data collection	HiSeq Illumina software
Data analysis	QIIME2 software with the available Online pipeline suitable for the Fastq files, Lefse with available tutorial pipeline.

For manuscripts utilizing custom algorithms or software that are central to the research but not yet described in published literature, software must be made available to editors and reviewers. We strongly encourage code deposition in a community repository (e.g. GitHub). See the Nature Research guidelines for submitting code & software for further information.

Data

Policy information about availability of data

All manuscripts must include a data availability statement. This statement should provide the following information, where applicable:

- Accession codes, unique identifiers, or web links for publicly available datasets
- A list of figures that have associated raw data
- A description of any restrictions on data availability

The sequence data have been deposited in the NCBI Sequence Read Archive under accession number PRJNA597453 (https://www.ncbi.nlm.nih.gov/sra/PRJNA597453) for BS, Dom and Sub naïve mice, and PRJNA635674 (https://www.ncbi.nlm.nih.gov/sra/PRJNA635674) for GF transplanted mice.

Field-specific reporting

Please select the one below that is the best fit for your research. If you are not sure, read the appropriate sections before making your selection.

☒ Life sciences ☐ Behavioural & social sciences ☐ Ecological, evolutionary & environmental sciences

For a reference copy of the document with all sections, see nature.com/documents/nr-reporting-summary-flat.pdf

Life sciences study design

All studies must disclose on these points even when the disclosure is negative.

Sample size	Sample size for the animals were determined based on commonly used sample size in gut microbiome studies in the literature. All the qualitative experiments that were performed the sample size was determined after performing a preliminary experiment to observe the SD and then experiments were designed accordingly.
Data exclusions	No data were excluded from the analyses.
Replication	In all the behavioral tests (DSR, FST and TCST) as well as the molecular tests (adipokines, RTPCR) and the gut microbiome 16S sequencing fro the Sib/Dom and GF transplanted mice, results of all analyses is presented including the SD between animal replicates.
Randomization	Randomization was performed in all animal experiments.
Blinding	Histology analyses were performed in complete blinding including numbering of the slides and quantification.

Reporting for specific materials, systems and methods

We require information from authors about some types of materials, experimental systems and methods used in many studies. Here, indicate whether each material, system or method listed is relevant to your study. If you are not sure if a list item applies to your research, read the appropriate section before selecting a response.

Materials & experimental systems

n/a	Involved in the study	
☐	☒	Antibodies
☒	☐	Eukaryotic cell lines
☒	☐	Palaeontology and archaeology
☐	☒	Animals and other organisms
☒	☐	Human research participants
☒	☐	Clinical data
☒	☐	Dual use research of concern

Methods

n/a	Involved in the study	
☒	☐	ChIP-seq
☒	☐	Flow cytometry
☒	☐	MRI-based neuroimaging

Antibodies

Antibodies used	Rabbit monoclonal [SP115] to F4/80, Abcam, catalog no.: ab111101, lot: GR3187118-2
Validation	https://www.abcam.com/f480-antibody-sp115-ab111101.html

Animals and other organisms

Policy information about studies involving animals; ARRIVE guidelines recommended for reporting animal research

Laboratory animals	Mice, SABRA, males and females, age: mostly adult (3 month), body weight follow up performed in 0-3 month. mice ages are detailed in each exp.
Wild animals	Study did not involved wild animals
Field-collected samples	The study did not involve samples collected from the field
Ethics oversight	Ariel University Institutional Ethical Committee

Note that full information on the approval of the study protocol must also be provided in the manuscript.
